# Cost-effectiveness of insulin degludec versus insulin glargine U100 in adults with type 1 and type 2 diabetes mellitus in Bulgaria

**DOI:** 10.1186/s12902-019-0460-6

**Published:** 2019-12-03

**Authors:** Monika Russel-Szymczyk, Vasil Valov, Alexandra Savova, Manoela Manova

**Affiliations:** 1 Novo Nordisk Pharma Sp. z o.o, Warsaw, Poland; 2 Novo Nordisk Pharma EAD, Sofia, Bulgaria; 3 Faculty of Pharmacy, Medical University Sofia, Sofia, Bulgaria

**Keywords:** Cost-effectiveness, Diabetes, Hypoglycaemia, ICER, Insulin degludec, QALY

## Abstract

**Background:**

This analysis evaluates the cost-effectiveness of insulin degludec (degludec) versus biosimilar insulin glargine U100 (glargine U100) in patients with type 1 (T1DM) and type 2 diabetes mellitus (T2DM) in Bulgaria.

**Methods:**

A simple, short-term model was used to compare the treatment costs and outcomes associated with hypoglycaemic events with degludec versus glargine U100 in patients with T1DM and T2DM from the perspective of the Bulgarian National Health Insurance Fund. Cost-effectiveness was analysed over a 1-year time horizon using data from clinical trials. The incremental cost-effectiveness ratio (ICER) was the main outcome measure.

**Results:**

In Bulgaria, degludec was highly cost-effective versus glargine U100 in people with T1DM and T2DM. The ICERs were estimated to be 4493.68 BGN/quality-adjusted life year (QALY) in T1DM, 399.11 BGN/QALY in T2DM on basal oral therapy (T2DM_BOT_) and 7365.22 BGN/QALY in T2DM on basal bolus therapy (T2DM_B/B_), which are below the cost-effectiveness threshold of 39,619 BGN in Bulgaria. Degludec was associated with higher insulin costs in all three patient groups; however, savings from a reduction in hypoglycaemic events with degludec versus glargine U100 partially offset these costs. Sensitivity analysis demonstrated that the results were robust and largely insensitive to variations in input parameters. At a willingness-to-pay threshold of 39,619 BGN/QALY, the probability of degludec being cost-effective versus glargine U100 was 60.0% in T1DM, 99.4% in T2DM_BOT_ and 91.3% in T2DM_B/B_.

**Conclusion:**

Degludec is a cost-effective alternative to biosimilar glargine U100 for patients with T1DM and T2DM in Bulgaria. Degludec could be of particular benefit to those patients suffering recurrent hypoglycaemia and those who require additional flexibility in the dosing of insulin.

## Background

The treatment of diabetes and its complications represents a major economic burden to healthcare systems worldwide. An estimated 425 people aged 20–79 years worldwide had diabetes in 2017 with an associated healthcare expenditure of approximately $727 billion [1]. This is projected to reach $776 billion by 2045. The mean diabetes-related expenditure in Bulgaria per person with diabetes is $798 per year [1]. The treatment of diabetes-related complications, such as cardiovascular disease, retinopathy and neuropathy, makes up the largest part of the direct medical costs associated with diabetes care, with less than 10% spent on insulin and anti-diabetic drugs [2, 3]. Diabetes is also associated with considerable indirect costs (e.g. due to absenteeism and lost productivity) [4].

The goal of diabetes treatment is to achieve good glycaemic control to prevent or delay macro- and microvascular complications and reduce cardiovascular and all-cause mortality [5, 6]. All people with type 1 diabetes (T1DM) require insulin. The Bulgarian Society of Endocrinology recommends the use of intensive insulin therapy with a basal-bolus regimen for people with T1DM [7]. Type 2 diabetes (T2DM) is a progressive disease and although glycaemic control can initially be achieved with the use of other anti-hyperglycaemic agents, a large proportion of people will eventually require insulin to achieve glycaemic targets [8]. The Bulgarian Society of Endocrinology guidelines recommend a patient-centred approach, and indicate that the treatment should be individually adjusted to patient age, disease progression, existing co-morbidities and preference [7]. Insulin is the most effective therapy for reducing blood sugar [8, 9], however, despite clear guidelines [10, 11], glycaemic control remains sub-optimal (HbA_1c_ > 7%) in a substantial number of patients [12–15]. In Bulgaria, approximately 50% of patients with T2DM in specialist care and 57% of patients in primary care have HbA_1c_ ≥ 6.5%, and for many patients regular testing and subsequent treatment adjustment is uncommon [16]. Insulin is often underutilised, as it is not initiated in a timely manner, titrated properly, or intensified appropriately [17, 18]. Fear of hypoglycaemia, weight gain and restrictive treatment regimens are key impediments to insulin use [19].

Insulin degludec (degludec) is a basal insulin with a duration of action of more than 42 h, and a distinct, slow absorption mechanism, which results in a flat and stable action profile [20, 21]. Degludec has a four times lower day-to-day variability in glucose-lowering effect compared with insulin glargine (glargine) U100 and U300 [22–24]. Degludec’s stable and long action profile allows for flexibility in the timing of insulin administration and allows people to advance or delay administration with no impact on short-term glycaemic control and minimal risk of hypoglycaemia [25].

A large scale clinical trial programme (BEGIN), which included more than 9000 people with T1DM and T2DM and spanned the entire treatment spectrum of insulin treatment for T1DM and T2DM, supports the efficacy and safety of degludec [26]. Based on meta-analyses, degludec mediates equivalent reductions in HbA_1c_ with a lower risk of hypoglycaemia compared with glargine U100 at a significantly lower total daily insulin dose in T1DM and T2DM with basal-only insulin [27, 28]. These results have been confirmed in real-world studies demonstrating better glycaemic control and fewer episodes of hypoglycaemia in patients switching from glargine U100 or other basal insulins to degludec [29, 30].

With the ever-increasing constraints on the healthcare budget, new interventions should represent good value for money. Understanding both the economic and clinical impact of an intervention helps decision makers determine resource use and optimal care for patients. In cost-effectiveness analyses, the value of interventions is estimated by comparing the relative cost and outcomes. The relative differences are presented as incremental cost-effectiveness ratio (ICER), which is the difference of costs of two interventions divided by the difference in health effects. Quality-adjusted life years (QALYs) are a measure that combines life expectancy and health-related quality of life and are an accepted measure of effectiveness [31]. Decision-makers consider the incremental cost per additional QALY gained when allocating healthcare resources, to achieve maximal economic and clinical benefits. Many countries define a financial threshold of acceptable cost-effectiveness. In Bulgaria, the commonly accepted threshold is 3x the gross domestic product (GDP) per capita (39,619 BGN/QALY), in line with World Health Organization recommendations [32].

Recent cost-effectiveness analyses have demonstrated that degludec is cost-effective versus glargine U100 (Lantus®) in the United Kingdom and Serbia [33, 34]. Biosimilar insulin glargine U100 (Abasaglar®) has recently entered the basal insulin analogue market in Bulgaria, expanding therapeutic options for patients. There are currently no head-to-head data of degludec versus Abasaglar®; however, cost-effectiveness analyses can be conducted using available data and plausible assumptions.

The objective of this study was to assess the cost-effectiveness of degludec versus biosimilar glargine U100 in the treatment of adults with T1DM and T2DM in Bulgaria from the perspective of the Bulgarian National Health Insurance Fund (NHIF).

## Methods

### Model overview

This cost-effectiveness model compared degludec with glargine U100 in three separate patient groups: T1DM using basal-bolus therapy (T1DM_B/B_); T2DM using basal-oral therapy (T2DM_BOT_); T2DM using basal-bolus therapy (T2DM_B/B_). Biosimilar glargine U100 (Abasaglar®) was chosen as the most appropriate comparator in the economic analyses as glargine is the most widely used basal insulin analogue in Bulgaria and biosimilar glargine U100 serves as reference price per daily defined dose (DDD).

The analysis assumes that Abasaglar® has the same efficacy and safety as originator glargine U100 (Lantus®).

**Fig. 1:**
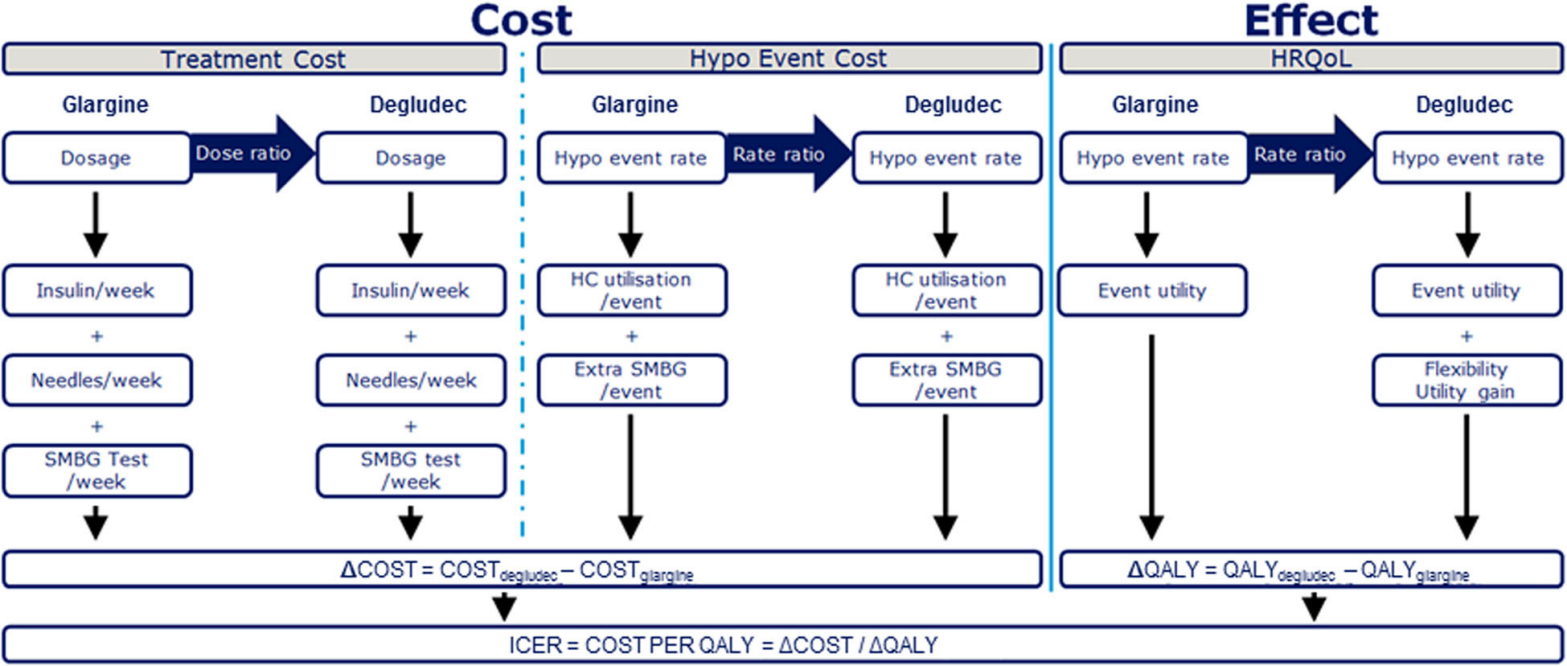
Overview of cost-effectiveness model. Abbreviations: Δ, change in; HC, healthcare, HRQoL, health-related quality of life; ICER, incremental cost-effectiveness ratio; QALY, quality-adjusted life year; SMBG, self-monitored blood glucose

For the current cost-effectiveness analysis, a simple model with a 1-year time horizon model was developed in Microsoft® Excel 2010 (Microsoft Corp., Redmond, WA, US) to capture the direct medical costs associated with insulin treatment and hypoglycaemia (Fig. 1). The effectiveness (QALY) was calculated by multiplying the disutility per hypoglycaemic event by the number of events in each treatment group.

The short-term model is appropriate as the treat-to-target trial design enforces a similar level of glycaemic control across comparators and no difference in terms of glycaemic control (difference in HbA_1c_) is expected. Therefore, there is no rationale for long-term modelling based on reductions in HbA_1c_. Although the cost-effectiveness of degludec was analysed in a short-term setting and based on data from 1-year clinical studies, results are not only applicable for the cost-effectiveness of degludec in the first year of treatment. As the model can be replicated for subsequent years, the outcomes represent the average annual cost-effectiveness in a steady state. As the time horizon was 1 year, no discounting was applied.

### Clinical data

#### Insulin dose

Units of insulin used per day for glargine U100 were taken from daily insulin doses used in clinical practice in Bulgaria [35]. The degludec/glargineU100 dose ratio was derived from a meta-analysis of insulin dose [27]. The degludec dose was derived from the dose ratio so that adjustment of covariate factors such as trial, treatment, anti-diabetic therapy at screening, age, sex, region, and baseline dose could be performed (Table 1). The insulin dose and dose ratio was solely used to calculate costs and was not included as a clinical outcome.

**Table 1:** Basal and bolus insulin use

Treatment group	Observed glargine U100 (units/day)	Dose ratio (degludec/glargine U100)	Calculated degludec (units/day)
T1DM_B/B_, total dose		0.88*	
Basal insulin	28.11	0.87*	24.46
Bolus insulin	37.13	0.88*	32.67
T2DM_BOT_, total dose		0.90*	
Basal insulin	28.11	0.90*	25.30
Bolus insulin	Not relevant	Not relevant	Not relevant
T2DM_B/B_, total dose		Not significant	
Basal insulin	28.11	1.08*	30.36
Bolus insulin	37.13	Not significant	37.13

Abbreviations: *B/B* Basal-bolus, *BOT* Basal oral therapy, *T1DM* Type 1 diabetes mellitus, *T2DM* Type 2 diabetes mellitus

* *p* < 0.05; *NS* Non-significant; in the case of non-significant results, a relative rate of one was used in the calculation

#### Hypoglycaemia event rates

Real-world non-severe hypoglycaemic event rates were derived from a large-scale multi-national survey of patients and physicians, and severe event rates were derived from a published observational study from the UK Hypoglycaemia Study Group (UKHSG) [36, 37]. Rates derived from observational studies more closely reflect real life event rates than clinical trial data, which can be biased in the selection of patients and the treatment setting. The base-case event rates in the glargine U100 group were the real-world hypoglycaemic event rates. Event rates for the degludec group were calculated using the degludec/glargine U100 hypoglycaemia rate ratios derived from a meta-analysis of hypoglycaemia which are adjusted for trial, type of diabetes, treatment, anti-diabetic therapy at screening, sex, region and age [27]. Only significant results were used for the calculation; in case of non-significant results, a rate ratio of one was used in the calculation (Table 2).

**Table 2:** Calculation of hypoglycaemia event rates

	Non-severe hypoglycaemia		Severe hypoglycaemia
	Daytime	Nocturnal	
T1DM			
Total events/patient/year for glargine U100^a^	30.42	8.52	3.20
Degludec/glargine U100 hypoglycaemic event rate ratio^b^	Not Significant	0.83*	Not significant
Calculated degludec hypoglycaemic event rate	30.42	7.07	3.20
T2DM_BOT_			
Total events/patient/year for glargine U100^a^	23.12	13.38	0.10
Degludec/glargine U100 hypoglycaemic event rate ratio^b^	Not significant	0.64*	0.14*
Calculated degludec hypoglycaemic event rate	23.11	8.57	0.01
T2DM_B/B_			
Total events/patient/year for glargine U100^a^	30.42	8.52	0.70
Degludec/glargine U100 hypoglycaemic event rate ratio^b^	0.83*	0.75*	Not significant
Calculated degludec hypoglycaemic event rate	25.25	6.39	0.70

Abbreviations: *B/B* Basal-bolus, *BOT* Basal oral therapy, *T1DM* Type 1 diabetes mellitus, *T2DM* Type 2 diabetes mellitus.

* *p* < 0.05; *NS* Non-significant; in the case of non-significant results, a relative rate of one was used in the calculation

^a^ Taken from Brod et al. [37] and UK Hypoglycaemia Study Group [36]

^b^ Taken from Vora et al. [27]

### Perspective

All costs and resources used were estimated from a healthcare payer perspective of the Bulgarian NHIF.

### Data used in the model

#### Direct treatment costs

The cost of insulin was calculated based on the pharmacy selling price (pharmacy purchase price, including VAT) in 2018, in BGN. The cost of needles and self-monitoring of blood glucose (SMBG) tests are not reimbursed by NHIF and are paid out of pocket by patients.

#### Cost of Hypoglycaemia

The methods used to derive hypoglycaemia cost (summarised in Table 3) have been reported previously [34]. It was assumed that the cost of treating a severe or non-severe hypoglycaemic event (diurnal and nocturnal) was similar regardless of whether the event was experienced by patients with T1DM, T2DM_BOT_ or T2DM_B/B_.

**Table 3:** Total costs of an average severe/non-severe hypoglycaemia event

	Cost per hypoglycaemic event in patients with T1DM, T2DM_BOT_, T2DM_B/B_		
	Non-severe daytime	Non-severe nocturnal	Severe
TOTAL (BGN)/event (2014)	0.66	33.60	514.36
TOTAL (BGN)/event (inflated to 2018)	0.65	33.19	508.10

Abbreviations: *B/B* Basal-bolus, *BGN* Bulgarian LEV, *BOT* Basal oral therapy, *T1DM* Type 1 diabetes mellitus, *T2DM* Type 2 diabetes mellitus

### Utility data

The methods used to derive utility data have been reported previously [34].

### Sensitivity analyses

One-way and probabilistic sensitivity analyses were conducted to assess the impact of varying key assumptions and outcomes used in the base case analysis. (Table 4).

**Table 4:** Sensitivity analyses conducted

Parameter	Base case	Sensitivity analysis
Time horizon	1 year	The time horizon was increased to 5 years.
Hypoglycaemia rates	Published literature: Brod et al. [37] UK Hypoglycaemia Study Group [36] Vora et al. [27]	Additional published event rates [27, 28, 38, 39] were investigated, including those reported in the clinical trial programme.
Mortality incidence after severe hypoglycaemia	1.12% mortality risk	The mortality risk was decreased to zero.
Cost of hypoglycaemia	Derived from clinical trial programme [40]	Healthcare costs were increased and decreased by 10%.
Insulin dose	Published literature: Vora et al. [27] Doneva et al. [35]	A sensitivity analysis was conducted assuming no difference in insulin dose between the treatments.
Flexible dosing utility	0.006 (Boye et al. [41])	Several sensitivity analyses were conducted to assess the impact of flexible dosing with degludec: one using an alternative utility value for flexible dosing (0.0130 [42]); one where only 50% of patients gained a utility benefit of flexible dosing; and one where there was no utility gain from dosing flexibility.

## Results

### Costs

The total costs for the three treatment groups are presented in Table 5. In T1DM, the total costs were estimated to be 3143.28 BGN per patient per year in the degludec group and 3073.92 BGN per patient per year for glargine U100, with approximately 40% of costs attributable to insulin and the remainder primarily due to severe hypoglycaemia. Total costs were 69.37 BGN higher (2.3% higher) in the degludec group than the glargine U100 group, which is due to the increased insulin costs with degludec, partially offset (47.19 BGN) by lower costs of hypoglycaemia due to a significantly lower rate of non-severe nocturnal hypoglycaemia versus glargine U100.

**Table 5:** Total cost per patient per year and incremental cost-effectiveness

	T1DM			T2DM_BOT_			T2DM_B/B_		
	Degludec (BGN/year)	Glargine U100 (BGN/year)	Incremental cost (BGN/year)	Degludec (BGN/year)	Glargine U100 (BGN/year)	Incremental cost (BGN/year)	Degludec (BGN/year)	Glargine U100 (BGN/year)	Incremental cost (BGN/year)
Insulin	1296.67	1180.11	116.56	794.93	574.23	220.70	1574.88	1196.94	377.95
Hypoglycaemia events	1846.61	1893.80	−47.19	306.40	509.74	− 203.34	581.79	655.53	−73.74
Non-severe daytime events	19.42	19.42	0.00	15.02	15.02	0.00	16.35	19.69	−3.35
Non-severe nocturnal events	230.41	277.60	−47.19	284.26	443.94	− 159.68	211.17	281.56	−70.39
Severe events	1596.78	1596.78	0.00	7.11	50.78	−43.67	354.28	354.28	0.00
Total costs	3143.28	3073.92	69.37	1101.32	1083.97	17.35	2156.67	1852.47	304.21
QALYs	0.5722	0.5568	0.0154	0.7490	0.7055	0.0435	0.6893	0.6480	0.0413
ICER (cost/QALY)	4498.68			399.11			7365.22		

Abbreviations: *B/B* Basal-bolus, *BGN* Bulgarian Lev, *BOT* Basal oral therapy, *ICER* Incremental cost-effectiveness ratio, *QALY* Quality-adjusted life years, *T1DM* Type 1 diabetes mellitus, *T2DM* Type 2 diabetes mellitus

In T2DM_BOT_, the total costs per patient per year were 1.6% higher in the degludec group (1101.32 BGN) versus the glargine U100 group (1083.97 BGN). The incremental costs were mainly driven by the increased cost of insulin, partially offset by lower costs of hypoglycaemia in the degludec group. Lower costs of hypoglycaemia were driven by significant reductions in the number of non-severe nocturnal and severe hypoglycaemic events in the degludec group versus glargine U100.

In the T2DM_B/B_ group, the total costs per person per year were 304.21 BGN (16.4%) higher in the degludec group (2156.67 BGN) versus the glargine U100 group (1852.47 BGN). The incremental costs with degludec were caused by increased cost of insulin which were partially offset by lower costs of non-severe hypoglycaemia. In the clinical trials, the dose of basal insulin in the degludec treatment arm was higher than in the glargine U100 arm [27] which drives the higher incremental costs in this group.

### Incremental cost-effectiveness

The incremental costs per QALY gained with degludec versus glargine U100 was estimated at 4499 BGN, 399 BGN and 7365 BGN in T1DM, T2DM_BOT_ and T2DM_B/B_, respectively. In all three settings, degludec was highly cost-effective versus glargine U100 with the ICER values falling considerably below the cost-effectiveness threshold assumed for Bulgaria (39,619 BGN) (Table 5).

### Sensitivity analysis

One-way sensitivity analyses demonstrate that the results are robust and largely insensitive to variations in input parameters (Additional file 1: Table S1–Table S3). The parameter with most influence on the ICER was the rate of hypoglycaemia. In T1DM, degludec remained highly cost-effective versus glargine U100 in all scenarios tested. The ICERs ranged between 45 and 19,781 BGN/QALY gained, with hypoglycaemic event rates having most influence on the ICER. When the number of non-severe and severe hypoglycaemic events was increased to those reported by Ericsson et al. [38], the ICER decreased to 45 BGN/QALY gained. Conversely, when the lower hypoglycaemic event rates reported in clinical trials [27, 28] were used, the ICER increased to 5472 BGN/QALY gained. Assuming no difference in the number of hypoglycaemic events between degludec and glargine U100 had the greatest effect on cost-effectiveness, increasing the ICER to 19,781 BGN/QALY gained. However, even in this scenario, degludec remained cost-effective vs glargine U100.

In T2DM_BOT_, the favourable cost-effectiveness results were invariant to changes in most of the input parameters tested. ICERs ranged between 32 BGN/QALY and 36,739 BGN/QALY gained. Using a lower number of hypoglycaemic events as reported in the clinical trials [27, 28], resulted in an increase in the ICER to 24,817 BGN/QALY gained but degludec remained cost-effective versus glargine U100 in this scenario. Conversely, using higher rates for severe hypoglycaemic events reported by Ericsson et al. [38], degludec became dominant versus glargine U100. Assuming no difference in hypoglycaemic event rates between degludec and glargine U100 had the greatest impact on the ICER, which increased to 36,739 BGN/QALY gained.

In T2DM_B/B_, the ICER was stable to reasonable variations in input parameters and was below the commonly accepted threshold for cost-effectiveness in almost all scenarios tested. The ICER increased when lower non-severe hypoglycaemic event rates were assumed (ICER 8141 BGN/QALY gained) and when assuming no difference in the non-severe hypoglycaemic event rates between degludec and glargine U100 (ICER 13,826–15,236 BGN/QALY gained). The ICER exceeded the cost-effectiveness threshold when no difference in the hypoglycaemia rate ratios was assumed (63,239 BGN/QALY).

### Probabilistic sensitivity analysis

**Fig. 2:**
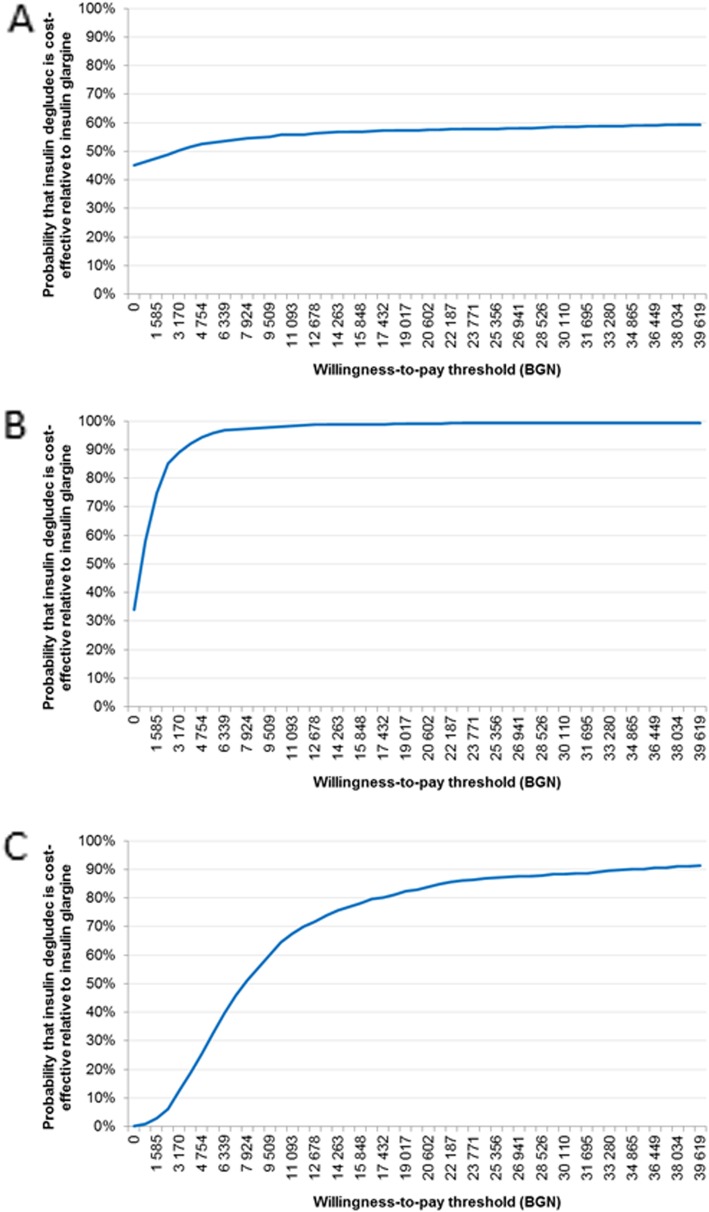
Probabilistic sensitivity analyses acceptability curves

To account for the uncertainty in the results caused by variation in data inputs, probabilistic sensitivity analyses were conducted. The cost-effectiveness acceptability curves demonstrated that, at a willingness-to-pay threshold of 39,619 BGN/QALY, the probability of degludec being cost-effective versus glargine U100 was 60.0% in T1DM, 99.4% in T2DM_BOT_ and 91.3% in T2DM_B/B_ (Fig. 2).

## Discussion

There are limited data on the cost-effectiveness of insulin treatments in Bulgaria. The number of insulin treatment options continues to increase, and decision making based on economic evidence is essential to optimise health outcomes while effectively managing limited budgets.

In this simple, short-term cost-effectiveness analysis, degludec was demonstrated to be cost-effective versus biosimilar glargine U100 in people with T1DM (ICER 4498.68 BGN), T2DM_BOT_ (ICER 399.11 BGN) and T2DM_B/B_ (ICER 7365.22 BGN). Although insulin costs with degludec were higher than with glargine U100, there is a reduction in the number of hypoglycaemic events with degludec in all three patient groups, which partially offsets the higher drug costs.

Cost-effectiveness analyses usually model the long-term impact of diabetes interventions on disease-related complications as a function of the differences in glycaemic control. However the data used in this model were derived from treat-to-target trials and glycaemic control was similar across both arms, thus the use of a long-term model based on differences in HbA_1c_ was not appropriate. Therefore, this simple, transparent short-term model focuses on other important aspects associated with insulin therapy, including hypoglycaemia and insulin dosing. Although this model only reflects a 1-year time horizon, it not only represents the cost-effectiveness of degludec vs glargine U100 within the first year of treatment, but it can also be replicated for subsequent years, representing annual cost-effectiveness. This is supported by the insensitivity of the ICER to changes in the time horizon in the sensitivity analyses. The model has previously been used to evaluate the cost-effectiveness of degludec versus glargine U100 in patients with T1DM and T2DM in different settings [33, 34, 38, 43–45]. In the United Kingdom (UK) and Denmark, degludec was found dominant versus glargine U100 in T1DM and T2DM_BOT_ and highly cost-effective in T2DM_B/B_ [33, 43]. Similarly, in Serbia and Sweden, degludec was cost-effective versus glargine U100 in all three patient groups [34, 38]. These results are consistent with those observed in the current study.

Hypoglycaemia can have a major impact on patient’s quality of life and lead to significant psychological and physical morbidity and in severe cases death [46, 47]. Additionally, people with recurrent hypoglycaemic episodes are at risk of developing impaired awareness of hypoglycaemia which can cause patients to miss early symptoms and treat hypoglycaemia, increasing the risk of severe hypoglycaemic events [48]. A recent real world multi-national, non-interventional study assessed the prevalence of hypoglycaemia worldwide, including Bulgaria. In Eastern Europe the estimated overall annual rate of hypoglycaemic events was 66.9 per person per year in T1DM and 23.7 per person per year in T2DM [49]. The prevalence of nocturnal and severe hypoglycaemia were 9.8 and 4.5 per person per year in T1DM and 4.0 and 2.2 per person per year in T2DM. The nocturnal hypoglycaemic event rates in T1DM and severe events in T1DM and T2DM in Bulgaria are higher than those used in this model which, based on the impact of hypoglycaemic event rates demonstrated in the sensitivity analyses, suggests that degludec may be even more cost-effective in real world clinical practice in Bulgaria.

The real-world cost effectiveness of switching patients with T1DM from other basal insulins (glargine U100, insulin detemir and NPH insulin) to degludec has been investigated using the IQVIA CORE diabetes model from the perspective of the United Kingdom and Sweden [30, 50]. The CORE diabetes model is a lifetime Markov model predicting diabetes complications over time in patient populations representative of clinical practice and calculates the resulting economic impact. From both the UK and Swedish perspective, degludec was dominant versus other basal insulins, which was mainly driven by the significant reduction in HbA_1c_ and lower rates of hypoglycaemia with degludec [30, 50].

The initiation and intensification of insulin regimens is often hampered by hypoglycaemia and the fear of hypoglycaemia [51]. As a result of fear of hypoglycaemia, approximately 52% of people with T1DM and 41% of people with T2DM reduce their insulin dose following a hypoglycaemic event [52]. This compromises glycaemic control and puts patients at risk of serious long-term complications, such as cardiovascular disease, renal disease, retinopathy, neuropathy and amputations [53]. The unique pharmacological profile of degludec with a flat and stable action profile is associated with lower rates of hypoglycaemia [20, 22]. In phase 3a clinical trials comparing degludec with glargine U100, degludec demonstrated equivalent reductions in HbA_1c_ with significantly lower rates of hypoglycaemia. In T1DM, degludec was associated with a 17% lower rate of non-severe nocturnal events, while in T2DM_BOT_ and T2DM_B/B_, rates of non-severe nocturnal hypoglycaemia were decreased by 36 and 25%, respectively [27]. Additionally, the rate of non-severe daytime events was 17% lower with degludec in T2DM_B/B_, and the rate of severe event hypoglycaemia was 86% lower in T2DM_BOT_ [27, 28].

In the SWITCH 1 and SWITCH 2 trials (phase 3b), degludec achieved equivalent reductions in HbA_1c_ with a significantly lower rate of overall symptomatic and severe hypoglycaemia versus glargine U100 in people with T1DM and T2DM with an increased risk of hypoglycaemia [54, 55]. These trials are more representative of patients in regular clinical practice than the phase 3a clinical trials, which excluded patients with recurrent hypoglycaemia. Data from SWITCH trials have recently been used to demonstrate that degludec is a cost-effective alternative to glargine U100 in the UK [56]. Furthermore, the hypoglycaemia benefit of degludec has been confirmed in real-world studies which demonstrate that switching to degludec from other basal insulin regimens is associated with significantly improved glycaemic control and a reduction in the rate of non-severe and severe hypoglycaemic events in T1DM and T2DM [29, 30].

In Bulgaria, SMBG tests are not paid by the healthcare payer but are paid out-of-pocket by patients, and were therefore not considered in this analysis. Due to the long duration of action with a flat and stable action profile and the lower day-to-day variability versus glargine U100, fewer SMBG tests are needed for titration and maintenance with degludec [20, 22, 57]. Fewer SMBG tests would be cost saving for the patient, although this was not assessed in the present analysis. The long and stable action profile of degludec also allows for flexibility of dosing time without compromising efficacy or risk of hypoglycaemia [25]. This flexible dosing option may provide an additional benefit especially for those people who have difficulties adhering to their treatment regimens (e.g. shift workers, frequent travellers, patients requiring help with insulin injections). Here, an estimate of the utility benefit of flexible dosing with degludec was included in the analysis and a utility gain of 0.006 derived from the study by Boye et al. [41] was applied for degludec. This can be considered a conservative estimate as a large time trade-off study identified a utility gain of 0.016 associated with flexible dosing of basal insulin and 0.013 vs fixed basal-bolus regimens [42].

It is important to acknowledge the limitations associated with the current analysis. With biosimilar glargine U100 being a relatively new-to-market insulin, no head-to-head trial data are available comparing degludec vs biosimilar glargine U100 and the analysis was based on currently available data and plausible assumptions, and the results should be interpreted accordingly. The analysis used hypoglycaemic event rates derived from published economic analyses using this model [33]. This could have resulted in an underestimation of the cost-effectiveness of degludec vs glargine U100 in Bulgaria, as a recent study suggests that rates of hypoglycaemia in real-world clinical practice in Bulgaria may be higher [49]. Additionally, the data used in this model to inform hypoglycaemia rate ratios originate from meta-analyses of phase 3a clinical trials [27, 28] to increase the statistical power of the analyses and increase the reliability of the data, and assumes replication of such rates in clinical practice. Except for hypoglycaemia, adverse events are not considered in our analysis, as head-to-head clinical trials have shown that degludec and glargine have similar safety profiles [26]. Furthermore, the cardiovascular outcomes trial DEVOTE showed that insulin degludec was non-inferior to insulin glargine in terms of major adverse cardiovascular events [58]. The clinical trials which informed this analysis used a treat-to-target approach in which patients were titrated until glycaemic targets were reached. In clinical practice, glycaemic targets are often not met for a variety of reasons, including non-adherence, and missed follow-up appointments. However, sensitivity analyses demonstrate that the results are robust to a wide variation in parameters, supporting the validity of the results.

## Conclusion

This short-term cost-effectiveness analysis demonstrates that degludec is a cost-effective alternative to biosimilar glargine U100 for patients with T1DM and T2DM in Bulgaria. Degludec could be of particular benefit to those patients suffering recurrent hypoglycaemia and those who require additional flexibility in the dosing of insulin.

## Supplementary information

**Additional file 1: Table S1.** Sensitivity analyses for T1DM, **Table S2.** Sensitivity analyses for T2DM_BOT_, **Table S3.** Sensitivity analyses for T2DM_B/B_.

## Data Availability

All data on which the conclusions of the manuscript are based are presented in the main paper or additional supporting files.

## Abbreviations

BGN: Bulgarian LEV
CI: Confidence interval
DDD: Daily defined dose
GDP: Gross domestic product
HC: Healthcare
HRQoL: Health-related quality of life
ICER: Incremental cost-effectiveness ratio
NHIF: National Health Insurance Fund
NS: Non-significant
QALY: Quality-adjusted life year
SMBG: Self-monitored blood glucose
T1DM: Type 1 diabetes mellitus
T1DMB/B: T1DM using basal-bolus therapy
T2DM: Type 2 diabetes mellitus
T2DMB/B: T2DM using basal-bolus therapy
T2DMBOT: T2DM using basal-oral therapy
TTO: Time trade off
