# Supplementary material for: Cost-effectiveness of insulin degludec versus insulin glargine U100 in adults with type 1 and type 2 diabetes mellitus in Bulgaria
Source: BMC Endocr Disord. 2019 Dec 3;19:132. doi: 10.1186/s12902-019-0460-6 (PMC6891960; doi:10.1186/s12902-019-0460-6)
Supplement: Supplementary file 1 — Additional file 1: Table S1. Sensitivity analyses for T1DM, Table S2. Sensitivity analyses for T2DMBOT, Table S3. Sensitivity analyses for T2DMB/B. [file 12902_2019_460_MOESM1_ESM.docx]

Table S1. Sensitivity analyses for T1DM

|  | **Sensitivity analyses** | **ΔCosts** | **ΔQALY** | **ICER** |
| --- | --- | --- | --- | --- |
| **Base case** |  | 69.37 | 0.0154 | 4,499 |
| Time horizon **Base case**: 1 year | 5 years | 304.77 | 0.0677 | 4,499 |
| Glargine U100 hypoglycaemia rates (events per 100 patient years) **Base case:**  Non-severe daytime: 3,041.67  Non-severe nocturnal: 851.67  Severe: 320.00 | Ostenson et al. 2014 [41]  Non-severe daytime: 7,098  Non-severe nocturnal: 2,002  Severe: 70 | 5.70 | 0.0287 | 199 |
|  | Ericsson et al 2013 [42]  Non-severe daytime: 8,528  Non-severe nocturnal: 2,080  Severe: 86 | 1.32 | 0.0295 | 45 |
|  | Vora et al. 2014 [27]  Non-severe daytime: 4,462  Non-severe nocturnal: 723  Ratner et al. 2013 [28]  Severe: 35 | 77.74 | 0.0142 | 5,472 |
| Degludec hypoglycaemia rate ratios  **Base case:** Non-severe daytime: 1.00  Non-severe nocturnal: 0.83  Severe: 1.00 | No difference in hypoglycaemia rates  Non-severe daytime: 1.00  Non-severe nocturnal: 1.00  Severe: 1.00 | 116.56 | 0.0059 | 19,781 |
| Non-severe hypoglycaemia healthcare costs  **Base case:**  Glargine U100  Daytime: 0.65  Nocturnal: 33.19  Degludec  Daytime: 0.65  Nocturnal: 33.19 | Increased by 10%  Glargine U100  Daytime: 0.72  Nocturnal: 36.51  Degludec  Daytime: 0.72  Nocturnal: 36.51 | 64.65 | 0.0154 | 4193 |
|  | Decreased by 10%  Glargine U100  Daytime: 0.59  Nocturnal: 29.21  Degludec  Daytime: 0.59  Nocturnal: 29.21 | 75.03 | 0.0154 | 4866 |
| Insulin dose  **Base case:**  Glargine U100  Basal: 28.1  Bolus: 37.1  Degludec:  Basal dose ratio: 0.87  Bolus dose ratio: 0.88 | Vora et al. 2014 [27]  Glargine U100  Basal: 33.1  Bolus: 35.0  Degludec:  Basal dose ratio: 0.87  Bolus dose ratio: 0.88 | 107.42 | 0.0154 | 6,966 |
|  | No difference in basal dose  Glargine U100  Basal: 28.1  Bolus: 37.1  Degludec:  Basal dose ratio: 1.00  Bolus dose ratio: 0.88 | 182.09 | 0.0154 | 11,809 |
|  | No difference in bolus dose  Glargine U100  Basal: 28.1  Bolus: 37.1  Degludec:  Basal dose ratio: 0.87  Bolus dose ratio: 1.00 | 143.20 | 0.0154 | 9,287 |
| Dosing flexibility  **Base case: 0.0060** | Evans et al. 2013 [43]: 0.0130 | 69.37 | 0.0223 | 3,111 |
|  | Only 50% of patients perceived benefit from flexibility: 0.0030 | 69.37 | 0.0125 | 5,561 |
|  | No utility associated with flexibility: 0.0000 | 69.37 | 0.0095 | 7,281 |

Abbreviations: ICER, incremental cost-effectiveness ratio; QALY, quality-adjusted life years; T1DM, type 1 diabetes mellitus.

Table S2. Sensitivity analyses for T2DM_BOT_

|  | **Sensitivity analyses** | **ΔCosts** | **ΔQALY** | **ICER** |
| --- | --- | --- | --- | --- |
| **Base case** |  | 17.35 | 0.0435 | 399 |
| Time horizon **Base case**: 1 year | 5 years | 83.38 | 0.2005 | 416 |
| Glargine U100 hypoglycaemia rates (events per 100 patient years) **Base case:**  Non-severe daytime: 2,311.67  Non-severe nocturnal: 1,338.33  Severe: 10.00 | Ostenson et al. 2014 [41]  Non-severe daytime: 1,380  Non-severe nocturnal: 650  Severe: 10 | 99.48 | 0.0269 | 3,696 |
|  | Ericsson et al 2013 [42]  Non-severe daytime: 1,404  Non-severe nocturnal: 884  Severe: 31 | -19.10 | 0.0435 | Dominant |
|  | Vora et al. 2014 [27]  Non-severe daytime: 154  Non-severe nocturnal: 51  Ratner et al. 2013 [28]  Severe: 2 | 205.66 | 0.0083 | 24,817 |
| Degludec hypoglycaemia rate ratios  **Base case:** Non-severe daytime: 1.00  Non-severe nocturnal: 0.64  Severe: 0.14 | No difference in hypoglycaemia rates  Non-severe daytime: 1.00  Non-severe nocturnal: 1.00  Severe: 1.00 | 220.31 | 0.0060 | 36,739 |
| Mortality incidence after severe hypoglycaemia  **Base case: 1.12%** | No difference in mortality incidence: 0% | 16.83 | 0.0431 | 390 |
| Non-severe hypoglycaemia healthcare costs  **Base case:**  Glargine U100  Daytime: 0.65  Nocturnal: 33.19  Degludec  Daytime: 0.65  Nocturnal: 33.19 | Increased by 10%  Glargine U100  Daytime: 0.72  Nocturnal: 36.51  Degludec  Daytime: 0.72  Nocturnal: 36.51 | 1.38 | 0.0435 | 32 |
|  | Decreased by 10%  glargine U100  Daytime: 0.59  Nocturnal: 29.21  Degludec  Daytime: 0.59  Nocturnal: 29.21 | 36.51 | 0.0435 | 840 |
| Severe hypoglycaemia healthcare costs  **Base case:**  Glargine U100: 508.1  degludec: 508.1 | Increase by 10%  glargine U100: 558.91  Degludec: 558.91 | 12.98 | 0.0435 | 299 |
|  | Decrease by 10%  glargine U100: 457.29  Degludec: 457.29 | 21.72 | 0.0435 | 500 |
| Insulin doses  **Base case:**  Glargine U100  Basal: 28.1  Degludec:  Basal dose ratio: 0.90 | Vora et al. 2014 [27]  Glargine U100  Basal: 51.7  Degludec:  Basal dose ratio: 0.90 | 202.56 | 0.0435 | 4,659 |
|  | No difference in basal dose  Glargine U100  Basal: 28.1  Degludec:  Basal dose ratio: 1.00 | 105.57 | 0.0435 | 2,428 |
| Dosing flexibility  **Base case: 0.0060** | Evans et al. 2013 [43]: 0.0130 | 17.35 | 0.0505 | 344 |
|  | Only 50% of patients perceived benefit from flexibility: 0.0030 | 17.35 | 0.0405 | 429 |
|  | No utility associated with flexibility: 0.0000 | 17.35 | 0.0375 | 463 |

Abbreviations: BOT, basal oral therapy; ICER, incremental cost-effectiveness ratio; QALY, quality-adjusted life years; T2DM, type 2 diabetes mellitus.

Table S3. Sensitivity analyses for T2DM_B/B_

|  | **Sensitivity analyses** | **ΔCosts** | **ΔQALY** | **ICER** |
| --- | --- | --- | --- | --- |
| **Base case** |  | 304.21 | 0.0413 | 7,365 |
| Time horizon **Base case**: 1 year | 5 years | 1372.64 | 0.1864 | 7,365 |
| Glargine U100 hypoglycaemia rates (events per 100 patient years) **Base case:**  Non-severe daytime: 3,041.67  Non-severe nocturnal: 851.67  Severe: 70.00 | Ostenson et al. 2014 [41]  Non-severe daytime: 2,761  Non-severe nocturnal: 779  Severe: 20 | 311.40 | 0.0382 | 8,141 |
|  | Ericsson et al 2013 [42]  Non-severe daytime: 3,692  Non-severe nocturnal: 884  Severe: 14 | 301.77 | 0.0465 | 6,489 |
| Degludec hypoglycaemia rate ratios  **Base case:** Non-severe daytime: 0.83  Non-severe nocturnal: 0.75  Severe: 1.00 | No difference in non-severe daytime hypoglycaemia rates  Non-severe daytime: 1.00  Non-severe nocturnal: 0.75  Severe: 1.00 | 307.56 | 0.0202 | 15,236 |
|  | No difference in non-severe nocturnal hypoglycaemia rates  Non-severe daytime: 0.83  Non-severe nocturnal: 1.00  Severe: 1.00 | 374.60 | 0.0271 | 13,826 |
|  | No difference in hypoglycaemia rates  Non-severe daytime: 1.00  Non-severe nocturnal: 1.00  Severe: 1.00 | 377.95 | 0.0060 | 63,239 |
| Non-severe hypoglycaemia healthcare costs  **Base case:**  Glargine U100  Daytime: 0.65  Nocturnal: 33.19  Degludec  Daytime: 0.65  Nocturnal: 33.19 | Increased by 10%  Glargine U100  Daytime: 0.72  Nocturnal: 36.51  Degludec  Daytime: 0.72  Nocturnal: 36.51 | 296.83 | 0.0413 | 7,187 |
|  | Decreased by 10%  Glargine U100  Daytime: 0.59  Nocturnal: 29.21  Degludec  Daytime: 0.59  Nocturnal: 29.21 | 312.99 | 0.0413 | 7,578 |
| Insulin doses  **Base case:**  Glargine U100  Basal: 28.1  Bolus: 37.1  Degludec:  Basal dose ratio: 1.08  Bolus dose ratio: 1.00 | Vora et al. 2014 [27]  Glargine U100  Basal: 66.6  Bolus: 72.7  Degludec:  Basal dose ratio: 1.10  Bolus dose ratio: 1.00 | 863.41 | 0.0413 | 20,904 |
|  | No difference in basal dose  Glargine U100  Basal: 28.1  Bolus: 37.1  Degludec:  Basal dose ratio: 1.00  Bolus dose ratio: 1.00 | 233.71 | 0.0413 | 5,658 |
| Dosing flexibility  **Base case: 0.0060** | Evans et al. 2013 [43]: 0.0130 | 304.21 | 0.0483 | 6,301 |
|  | Only 50% of patients perceived benefit from flexibility: 0.0030 | 304.21 | 0.0383 | 7,940 |
|  | No utility associated with flexibility: 0.0000 | 304.21 | 0.0353 | 8,611 |

Abbreviations: B/B, basal/bolus; ICER, incremental cost-effectiveness ratio; QALY, quality-adjusted life years; T2DM, type 2 diabetes mellitus.
